# Chromosome-level genome assembly of the largefin longbarbel catfish (*Hemibagrus macropterus*)

**DOI:** 10.3389/fgene.2023.1297119

**Published:** 2023-11-01

**Authors:** Huan Ye, Jiahui Fan, Yanling Hou, Huamei Yue, Rui Ruan, Shuang Li, Chongjiang Hu, Yong Xie, Chuangju Li

**Affiliations:** ^1^ Key Laboratory of Freshwater Biodiversity Conservation, Ministry of Agriculture and Rural Affairs, Yangtze River Fisheries Research Institute, Chinese Academy of Fishery Sciences, Wuhan, China; ^2^ Chongqing Fishery Sciences Research Institute, Chongqing, China

**Keywords:** *Hemibagrus macropterus*, genome assembly, genome annotation, comparative genomics, Hi-C, PacBio

## Abstract

The largefin longbarbel catfish, *Hemibagrus macropterus*, is an economically important fish species in southwestern China, with males growing faster than females. This study presents a high-quality chromosome-level genome assembly of the largefin longbarbel catfish, generated by integrating Illumina short reads, PacBio HiFi long reads, and Hi-C data. The assembled genome size was 858.5 Mb, with a contig and scaffold N50 of 5.8 Mb and 28.4 Mb, respectively. A total of 656 contigs were successfully anchored to 30 pseudochromosomes with a BUSCO score of 97.7%, consistent with the number of chromosomes analyzed by karyotype. The genome contained 29.5% repeat sequences, and a predicted total of 26,613 protein-coding genes, of which 25,769 (96.8%) were functionally annotated in different databases. Evolutionary analysis showed that *H. macropterus* was most closely related to *H. wyckioides*, with a divergence time of approximately 16.3 million years. Chromosomal syntenic relationships among *H. macropterus*, *H. wyckioides*, and *Pelteobagrus fulvidraco* revealed a one-to-one relationship for most chromosomes, except for break, fission, and inversion of some chromosomes. The first high-quality reference genome will not only provide a valuable genetic resource for the study of sex determination mechanisms and genetic breeding of largefin longbarbel catfish, but also contribute to comparative analyses of genome and chromosome evolution within Siluriformes.

## Introduction

Catfish (order: Siluriformes) are a highly diverse and globally distributed group of actinopterygian fish, generally characterized by the whisker-like barbels, lack scales, and intramuscular spines (Gisbert et al., 2022). They comprise more than 4,500 species and account for nearly 12% of teleost fish (Fricke et al., 2022). Catfish are one of the most important aquaculture species worldwide (Gisbert et al., 2022). The number of chromosomes in catfish ranges from 2n = 24 to 100, with mainly continuous variation from 2n = 48 to 60 (Zhu and Pan, 2021). Consequently, catfish are considered suitable for studying genomic and chromosomal evolution in fish. With the rapid development of sequencing technologies, the chromosome-level genomes of more than 10 catfish species have been assembled, including *Ictalurus punctatus* (Liu et al., 2016), *Pelteobagrus fulvidraco* (Gong et al., 2018), *Bagarius yarrelli* (Jiang et al., 2019), *Silurus meridionalis* (Zheng et al., 2021), *Leiocassis longirostris* (He et al., 2021), *Hemibagrus wyckioides* (Shao et al., 2021), *Pangasianodon hypophthalmus* (Gao et al., 2021), *Pseudobagrus ussuriensis* (Zhu et al., 2022), *Cranoglanis bouderius* (Xu et al., 2022), *Ictalurus furcatus* (Wang et al., 2022), and *Ancistrus triradiatus* (Lemopoulos and Montoya-Burgos, 2022). These genomic resources facilitate studies of sex determination mechanisms (Bao et al., 2019; Gong et al., 2022), chromosomal and genome evolution (Zhu et al., 2022), ecological adaptation, and gene evolution and function (Liu et al., 2016; Zhou et al., 2023).

**FIGURE 1:**
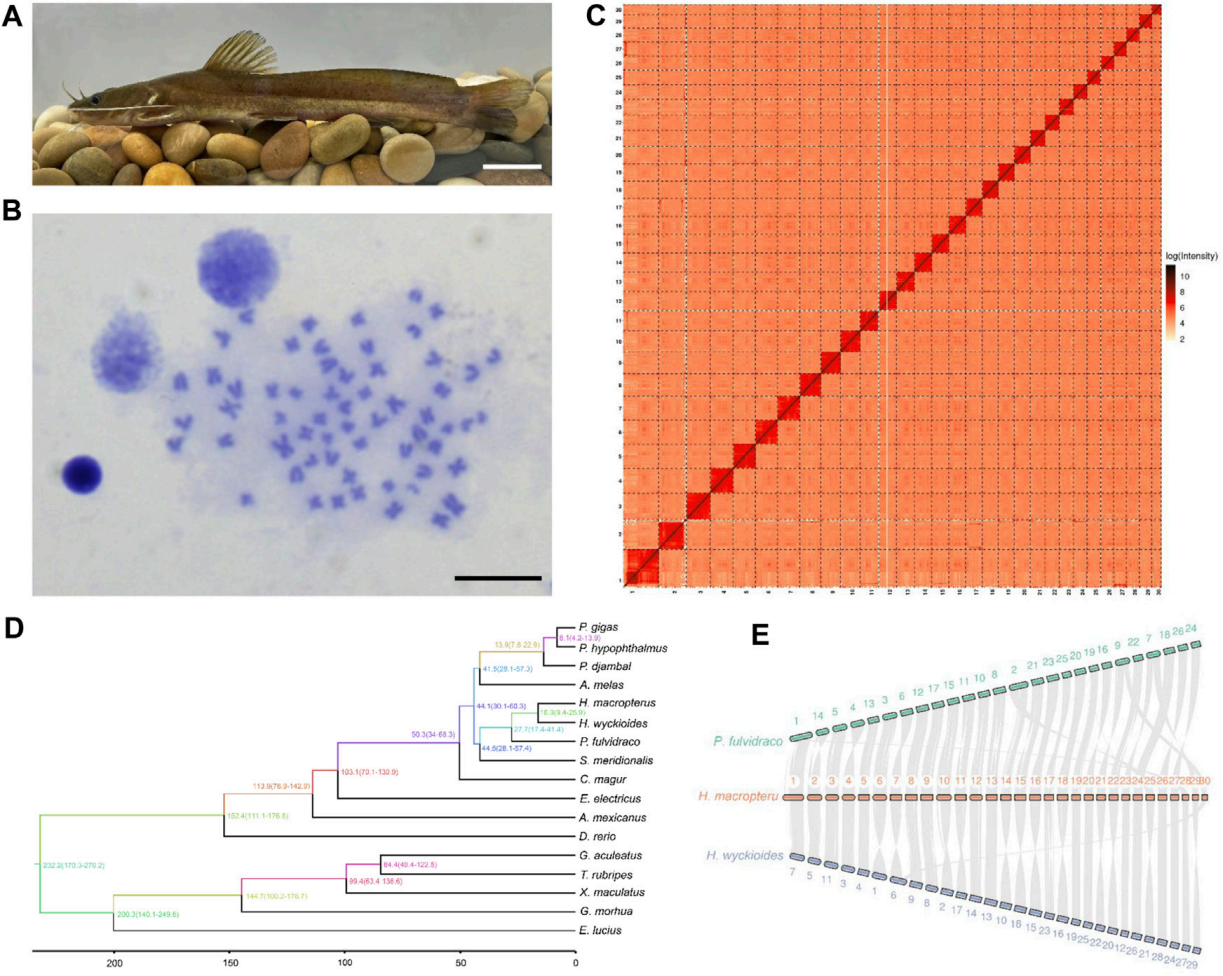
Chromosome-level genome assembly of *Hemibagrus macropterus* and comparative genomics analysis. **(A)** Representative image of *H. macropterus*. Scale bar represents 5 cm. **(B)** Karyotype of male *H. macropterus*. Scale bar represents 10 µm. **(C)** Heatmap of Hi-C interactions among 30 pseudochromosomes. Colour depth represents the density of the Hi-C interactions. **(D)** Phylogenetic relationships between *H. macropterus* and 16 other fish species based on 3,105 single-copy orthologous genes. **(E)** Chromosomal syntenic relationships among *H. macropterus*, *H. wyckioides*, and *Pelteobagrus fulvidraco*.

The largefin longbarbel catfish (*Hemibagrus macropterus*) (Figure 1A), belonging to the Bagridae family (Siluriformes), is an important commercial fish in southwestern China because of its high nutritional value (Zhang et al., 2009). It is a benthic dweller naturally distributed in the mainstream and tributaries of the Yangtze and Pearl River Basins (Zhu et al., 2007). Largefin longbarbel catfish exhibit sexual size dimorphism, with males growing faster than females. Two karyotypes (2n = 56 and 60) have been reported for this species among different populations (Hong and Zhou, 1984; Ma et al., 2013). However, the genetic resources of largefin longbarbel catfish are limited, which is unfavorable for understanding its genetic characteristics and developing breeding programs.

In this study, we present the first high-quality chromosome-level reference genome of *H. macropterus*. The assembled genome will be beneficial for exploring the genome evolution, sex determination mechanisms, and genetic breeding of largefin longbarbel catfish. Furthermore, this contribution to the genomic resources of Siluriformes will facilitate future comparative genomic studies among catfish.

## Data

### Genome assembly

A total of 31.4 Gb Illumina clean data were used to assess genome size and heterozygosity in *H. macropterus*. The predicted genome size was approximately 873.7 Mb and the estimated heterozygosity rate was 0.37%. For *de novo* genome assembly, 41.8 Gb PacBio HiFi reads were preliminarily assembled into 691 contigs with an N50 length of 5.8 Mb, covering 98.3% of the estimated genome. Using the Hi-C technique, a total of 656 contigs were successfully anchored to 30 pseudochromosomes (Figure 1C), consistent with the number of chromosomes analyzed by karyotype (Figure 1B) and reported in a previous study (Hong and Zhou, 1984). The assembled chromosome-level genome consisted of 35 contigs and 30 scaffolds, with a contig and scaffold N50 length of 5.8 and 28.4 Mb, respectively (Table 1), which represented 98.3% of the estimated genome. The guanine-cytosine (GC) content was 40%, similar with that of other Bagridae (Zhu et al., 2022). Benchmarking Universal Single-Copy Orthologs (BUSCO) analysis revealed 97.7% of BUSCO genes identified in the genome (Supplementary Table S1), indicating high completeness for the genome assembly.

**TABLE 1:** Statistics of *Hemibagrus macropterus* genome assembly and annotation.

Item	Category	Number
Sequencing data	PacBio HiFi (Gb)	41.8
	Illumina short WGS (Gb)	31.4
	Hi-C(Gb)	98.4
Assembly	Estimated genome size (Mb)	873.7
	Assembled genome size (Gb)	858.5
	Contig number	35
	Contig N50 (Mb)	5.8
	Scaffold number	30
	Scaffold N50 (Mb)	28.4
	Longest scaffold (Mb)	57.5
Annotation	GC content (%)	40.0
	Repeat sequences (%)	29.5
	Number of protein-coding genes	26,613
	Number of functional annotated genes	25,769
	Average gene length (bp)	25,071.3
	Average exon length (bp)	171.4
	Average intron length (bp)	2,880.7
	Average exon per gene	9.2

### Genome annotation

The genome of *H. macropterus* contained 29.5% repetitive sequences (Supplementary Table S2), with transposable element (TE) accounting for 18.06% of the assembled genome. The largest proportion of TE was terminal inverted repeats (9.29%), followed by long terminal repeat retrotransposons (7.43%). Together with homology, *de novo*, and RNA-seq prediction methods, 26,613 protein-coding genes were annotated (Supplementary Table S3). The average length of gene, exon, and intron was 25,071, 171, and 2,881 bp, respectively (Table 1). BUSCO assessments showed that 98.2% complete BUSCO genes were predicted, including 96.1% single copy and 2.1% duplicated genes (Supplementary Table S4). These results indicated high-quality genome assembly and annotation of *H. macropterus*.

### Genome evolution analysis

The evolutionary relationships between *H. macropterus* and other teleosts were determined based on the analysis of 3,105 single-copy orthologous genes from of 17 fish genomes (Supplementary Figure S1). *H. wyckioides* was most closely related to *H. macropterus*, consistent with their taxonomic relationship (Fricke et al., 2022), and clustered with *P. fulvidraco* (Figure 1D). The nine species of Siluriformes formed a monophyletic clade, and then together with *Electrophorus electricus* (Gymnotiformes), *Astyanax mexicanus* (Characiformes), and *Danio rerio* (Cypriniformes), formed the clade of Ostariophysan. According to the fossil calibration times, the estimated divergence time between *H. macropterus* and *H. wyckioides* was approximately 16.3 Mya, and the divergence time between *M. macropterus* and *P. fulvidraco* was around 27.7 Mya.

Through comparative genomic analysis, we identified 60 and 28 gene families, respectively, that underwent significant expansion and contraction in *H. macropterus* (Supplementary Figure S2). Enrichment analysis revealed that the expanded and contracted genes were enriched in 18 and 7 Kyoto Encyclopedia of Genes and Genomes (KEGG) pathways, respectively (Supplementary Tables S5, S6), with most involved in the immunity, metabolism, and hormone biosynthesis. These results provide valuable preliminary information on the biological properties of the species.

### Synteny anslysis

We compared the chromosome syntenies between *H. macropterus* and two other catfish species. The karyotype of these three species (*P. fulvidraco*, *H. macropterus*, and *H. wyckioides*) was 2n = 52, 2n = 60, and 2n = 59, respectively. Most of the chromosomes between *P. fulvidraco* and *H. macropterus* exhibited a one-to-one relationship (Figure 1E), whereas the chromosomes (Chr) 1, 2, 7, and 9 of *P. fulvidraco* broke into two chromosomes in *H. macropterus. H. macropterus* and *H. wyckioides* displayed a strong one-to-one correspondence among their chromosomes, except for the fission of *H. wyckioides* Chr6 into Chr7 and 30, and inversion of some chromosomes (Chr6, 7, 10, 11, 14, 15, 16, 18, 21, 22, 25, and 29) in *H. macropterus*. It was recently reported that the sex-determining region of *P. fulvidraco* and *H. wyckioides* was located on the Chr 2 and 26, respectively (Gong et al., 2022), whereas the correspondent chromosomes in *H. macropterus* were Chr14 and 15 and 24, respectively, indicating the complexity of the sex-determining region or chromosome in catfish.

## Materials and methods

### Sample collection and sequencing

A male *H. macropterus* was collected for genome sequencing from the Wuhan section of the Yangtze River. After anesthesia with 3-aminobenzoic acid ethyl ester methanesulfonate-222 (MS-222) (Sigma-Aldrich, St. Louis, MO, United States), muscle tissue was collected, frozen in liquid nitrogen, and stored at −80°C, while other tissues including brain, gills, heart, intestine, kidney, liver, spleen, and testis were stored in RNAlater solution (Sigma-Aldrich). All experiments involving in the handling and treatment of fish were conducted in accordance with the Guidelines for the Care and Use of Laboratory Animals of the Yangtze River Fisheries Research Institute, Chinese Academy of Fishery Sciences.

Total genomic DNA was isolated from frozen muscle tissue using a Blood & Cell Culture DNA Kit (Qiagen, Hilden, Germany). DNA quality and purity were determined using a Nanodrop 2000 (Thermo Fisher Scientific, Waltham, MA, United States) and agarose gel electrophoresis. A 350 bp paired-end library was constructed using an Illumina TruSeq DNA Nano Preparation Kit (Illumina, San Diego, CA, United States) and sequenced on an Illumina HiSeq 2500 platform (Illumina). Approximately 5 μg of genomic DNA was used to construct a PacBio SMRTbell library. The library was sequenced using a PacBio Circular Consensus Sequencing (CCS) Platform (PacBio, Menlo Park, CA, United States). A Hi-C library was prepared using a GrandOmics Hi-C kit according to the manufacturer’s instructions and sequenced on an Illumina NovaSeq platform (Illumina). Total RNA was extracted from different tissues of *H. macropterus* using an RNeasy Plus Mini Kit (Qiagen). A complementary DNA library was constructed using a TruSeq Stranded mRNA-Seq kit on the Illumina HiSeq 2500 platform (Illumina).

### Preparation of chromosome metaphases

Three male *H. macropterus* juveniles were injected twice with phytohemagglutinin (PHA) at 10 μg/g body weight with a 12 h interval, injected with colchicine at 10 μg/g body weight for 3 h, and anaesthetised using MS-222. Kidney cells were collected by hypotonic and fixation treatments as previously described (Zhu and Gui, 2007) and the number of mitotic metaphase chromosomes was counted in 100 cells.

### Genome assembly and evaluation

Illumina clean short reads were used to estimate genome size and heterozygosity based on k-mer frequency distribution analysis using Jellyfish (Marçais and Kingsford, 2011). The PacBio long reads were assembled *de novo* into contigs using Hifiasm (Cheng et al., 2021), and then polished using Illumina short reads and NextPolish (Hu et al., 2019). For chromosome-level assembly of the *H. macropterus* genome, clean Hi-C reads were mapped to the primary genome using Bowtie2 (Langmead and Salzberg, 2012). HiC-Pro was used to validate interacting paired reads (Servant et al., 2015). Primary assembly scaffolds were oriented, ordered, and clustered on pseudochromosomes using LACHESIS (Korbel and Lee, 2013). JuiceBox was used to adjust the placement and orientation errors (Durand et al., 2016), and a Hi-C heat map was constructed.

Two strategies were used to assess genome completeness. The BUSCO completeness score of the assembled genome was evaluated using the Actinopterygii database (Simão et al., 2015), and RNA-seq data were mapped back to the genome using HISAT2 with default settings (Kim et al., 2015).

### Genome annotation

Repetitive elements of the *H. macropterus* genome were annotated using both homology and *de novo* strategies. According to the structural features, tandem and simple sequence repeats were predicted using TRF (Benson, 1999) and MISA (Beier et al., 2017), respectively, with default parameters. The transposable elements were identified using LTR_Finder (Ou and Jiang, 2019), LTRharverst (Ellinghaus et al., 2008), and LTR_retriver (Ou and Jiang, 2017). *De novo* annotation of other repeat sequences was performed using RepeatModeler (Price et al., 2005), followed by genome-scale detection using RepeatMasker (Chen, 2004). The combined results of these two predictions provided the final annotation of the non-redundant repeat elements in the genome.

We combined *de novo*, homology, and transcriptome-based methods to predict protein-coding genes. For *de novo* prediction, we used Augustus (Keller et al., 2011), GlimmerHMM (Majoros et al., 2004), and Geneid (Blanco et al., 2007) with their default parameters. Protein sequences of *D. rerio*, *E. electricus*, *Esox lucius*, *Gadus morhua*, *H. wyckioides*, *Silurus meridionalis*, *P. fulvidraco*, and *Takifugu rubripes* were aligned to the genome of *H. macropterus* using TBLASTN (Camacho et al., 2009). GeneWise (Birney et al., 2004) was used to predict the gene structure according to homology alignments. For transcriptome-based prediction, protein-coding regions were identified by aligning the transcripts with the assembled genome using PASA (Haas et al., 2003). Transposons were removed using TransposonPSI, and the final non-redundant reference gene set was obtained using EVidenceModeler (Haas et al., 2008).

For the functional annotation, the gene set was aligned to proteins deposited in the SwissProt and NCBI non-redundant protein databases using BLASTP. KEGG pathways were annotated by the KEGG Automatic Annotation Server (Moriya et al., 2007). Gene Ontology and protein domains were identified using InterProScan (Jones et al., 2014) with default parameters.

### Phylogenetic and comparative genomic analyses

Orthologous gene families were identified by comparing the predicted protein sequences of H. macropterus with those of 16 other fish using OrthoMCL (Li et al., 2003). Single-copy gene orthogroups among these species were selected and aligned using MAFFT V7 (Yamada et al., 2016). A maximum likelihood phylogenetic tree was constructed using RAXML7 (Stamatakis, 2015) with 1,000 bootstrap replicates. Divergence time was estimated using MCMCTREE in PAML4 (Yang, 2007), and calibrated using fossil divergence times from the TimeTree database (http://www.timetree.org/). Based on the results of OrthoMCL, expanded and contracted gene families were analyzed via CAFE (De Bie et al., 2006), and functional enrichment analysis was performed by alignment homologues against the KEGG pathway database.

### Chromosomal syntenic analysis

To investigate the chromosomal syntenic relationships among *H. macropterus*, *H. wyckioides*, and *P. fulvidraco*, MCscan (Tang et al., 2008) was applied to determine the syntenic blocks. Proteomes were compared between the pairs of species using BLASTP with an e-value of 1e-5, and a minimum of four genes in each block were used for synteny calling.

## Data Availability

The datasets presented in this study can be found in online repositories. The names of the repository/repositories and accession number(s) can be found in the article/Supplementary Material.

## Supplementary material

SUPPLEMENTARY FIGURE S1 Comparison of the number of homologous genes present in the genomes of *Hemibagrus macropterus* and 16 other fish species.

SUPPLEMENTARY FIGURE S2 Expansion and contraction of *Hemibagrus macropterus* gene families. The number and sector with green, red, and blue represent the specific values of the gene families of expansion, contraction, and stability for each species, respectively. MRCA, most recent common ancestor.
