# Supplementary material for: Chromosome-level genome assembly of the largefin longbarbel catfish (Hemibagrus macropterus)
Source: Front Genet. 2023 Nov 1;14:1297119. doi: 10.3389/fgene.2023.1297119 (PMC10646426; doi:10.3389/fgene.2023.1297119)
Supplement: Supplementary file 1 [file Table1.docx]

**Supplementary Table S1.** Completeness of the *Hemibagrus macropterus* genome determined by BUSCO.

| Type | Number | Percent (%) |
| --- | --- | --- |
| Complete BUSCOs | 3,558 | 97.7 |
| Complete and single-copy BUSCOs | 3,488 | 95.8 |
| Complete and duplicated BUSCOs | 70 | 1.90 |
| Fragmented BUSCOs | 21 | 0.60 |
| Missing BUSCOs | 61 | 1.70 |
| Total BUSCO groups searched | 3,640 | 100.00 |
