# Supplementary figures and images for: Chromosome-level genome assembly of the largefin longbarbel catfish (Hemibagrus macropterus)
Source: Front Genet. 2023 Nov 1;14:1297119. doi: 10.3389/fgene.2023.1297119 (PMC10646426; doi:10.3389/fgene.2023.1297119)

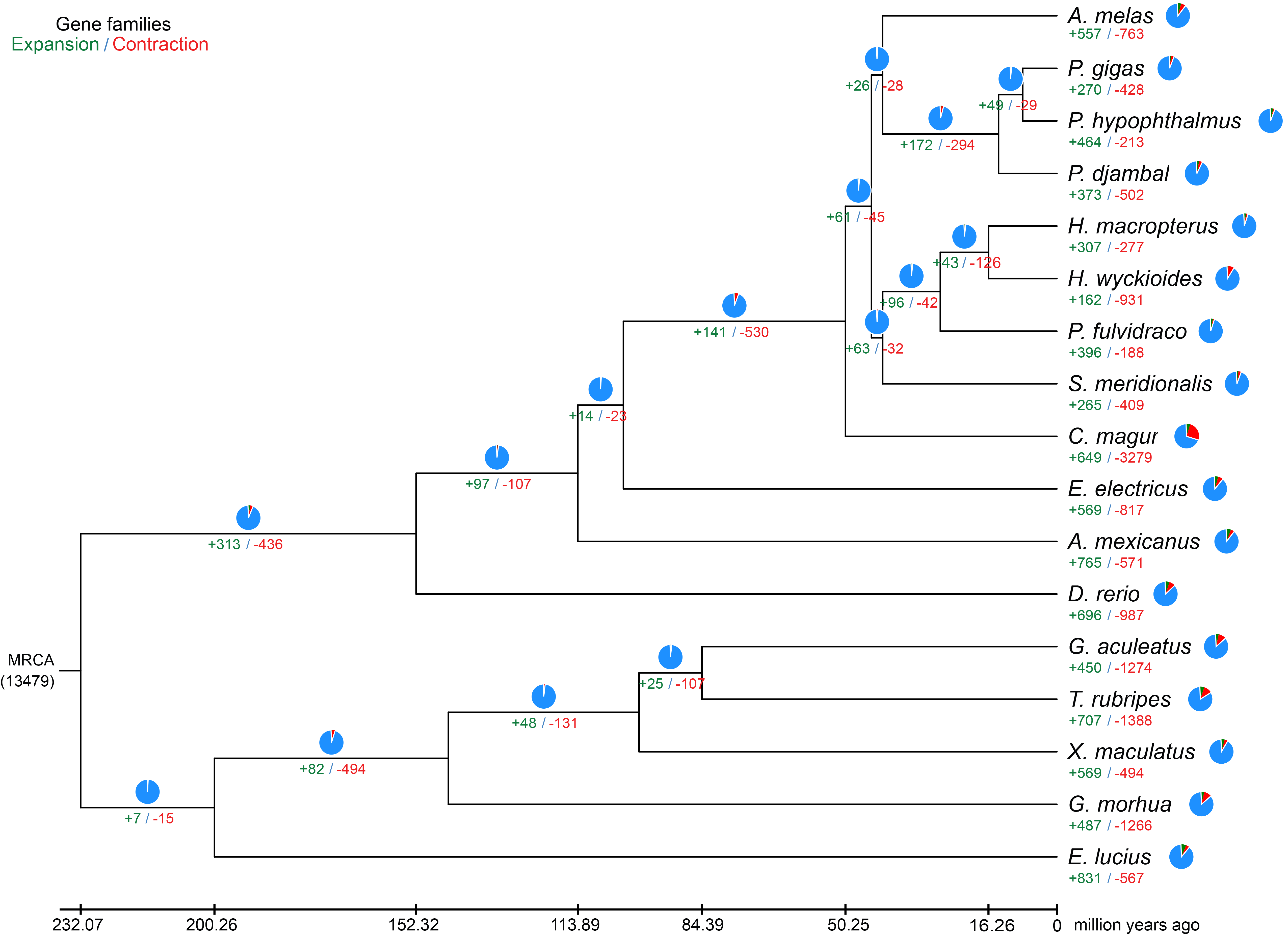

Supplement: Supplementary file 2 [file Image2.TIF]

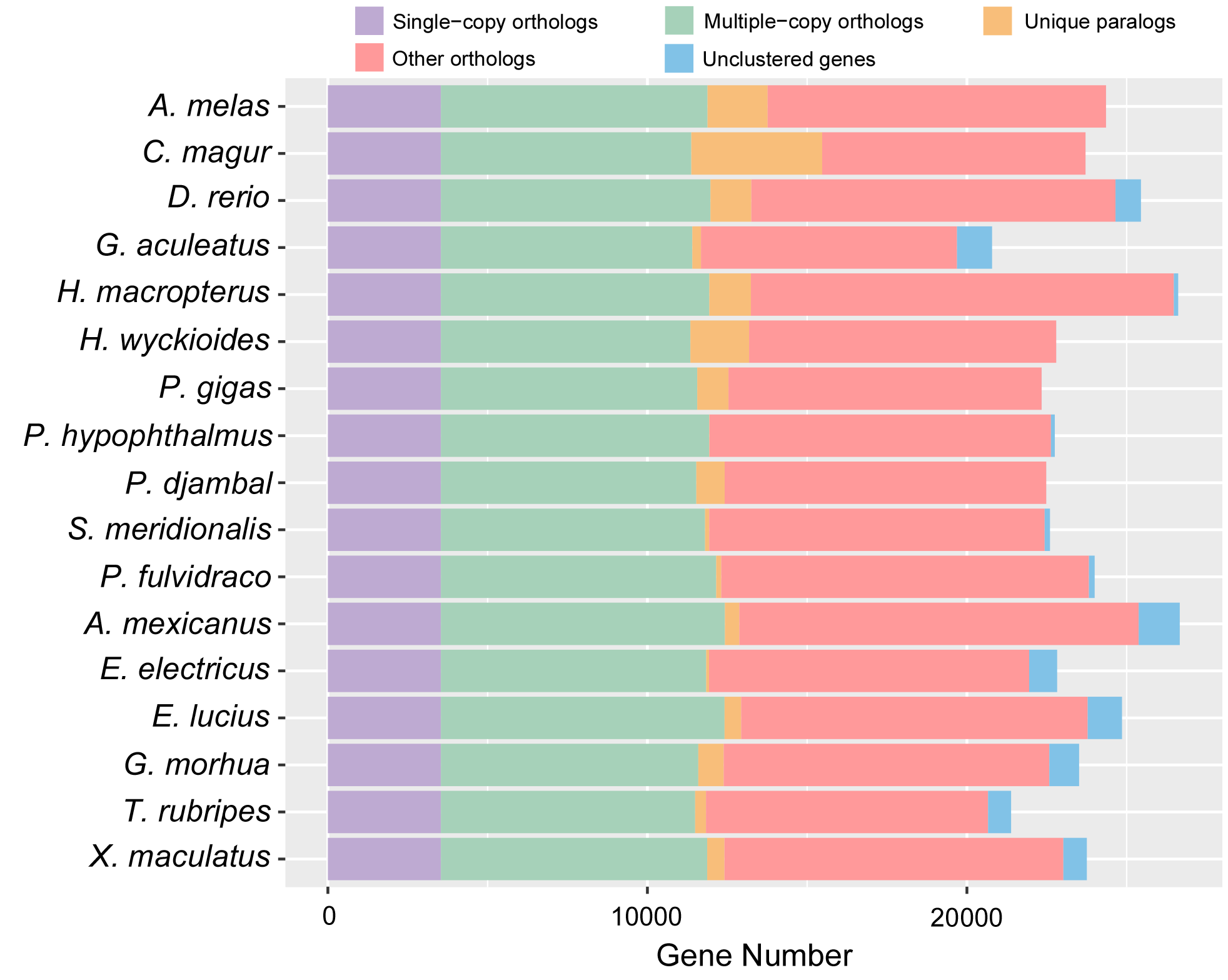

Supplement: Supplementary file 3 [file Image1.TIF]
