# Supplementary material for: Chromosome-level genome assembly of the largefin longbarbel catfish (Hemibagrus macropterus)
Source: Front Genet. 2023 Nov 1;14:1297119. doi: 10.3389/fgene.2023.1297119 (PMC10646426; doi:10.3389/fgene.2023.1297119)
Supplement: Supplementary file 4 [file Table2.docx]

**Supplementary Table S2.** Statistics of repeat sequences identified in the *Hemibagrus macropterus* gnome assembly.

| Type | Length (bp) | % in genome |
| --- | --- | --- |
| LTR | 63,833,814 | 7.43 |
| TIR | 79,773,780 | 9.29 |
| low_complexity | 18,299,398 | 2.13 |
| nonLTR | 1,547,996 | 0.18 |
| nonTIR | 9,927,535 | 1.16 |
| repeat_region | 80,040,628 | 9.32 |
| Total | 253,423,151 | 29.52 |
