# Supplementary material for: Chromosome-level genome assembly of the largefin longbarbel catfish (Hemibagrus macropterus)
Source: Front Genet. 2023 Nov 1;14:1297119. doi: 10.3389/fgene.2023.1297119 (PMC10646426; doi:10.3389/fgene.2023.1297119)
Supplement: Supplementary file 5 [file Table5.docx]

**Supplementary Table S5.** Expansion gene families of male *Hemibagrus macropterus* were enriched in 18 KEGG pathways.

| ID | Description |
| --- | --- |
| map05322 | Systemic lupus erythematosus |
| map05034 | Alcoholism |
| map05416 | Viral myocarditis |
| map04621 | NOD-like receptor signaling pathway |
| map05226 | Gastric cancer |
| map04062 | Chemokine signaling pathway |
| map00340 | Histidine metabolism |
| map00140 | Steroid hormone biosynthesis |
| map01523 | Antifolate resistance |
| map05216 | Thyroid cancer |
| map00480 | Glutathione metabolism |
| map04066 | HIF-1 signaling pathway |
| map04727 | GABAergic synapse |
| map05167 | Kaposi sarcoma-associated herpesvirus infection |
| map05417 | Lipid and atherosclerosis |
| map05135 | Yersinia infection |
| map04515 | Cell adhesion molecules |
| map04610 | Complement and coagulation cascades |
