# Supplementary material for: Chromosome-level genome assembly of the largefin longbarbel catfish (Hemibagrus macropterus)
Source: Front Genet. 2023 Nov 1;14:1297119. doi: 10.3389/fgene.2023.1297119 (PMC10646426; doi:10.3389/fgene.2023.1297119)
Supplement: Supplementary file 6 [file Table3.docx]

**Supplementary Table S3.** Statistics of the annotated protein-coding genes in the *Hemibagrus macropterus* genome.

| Type | | Number | Percent (%) |
| --- | --- | --- | --- |
| Annotation | SwissProt | 22,658 | 85.14 |
|  | KEGG | 24,060 | 90.41 |
|  | KOG | 15,489 | 58.20 |
|  | GO | 22,225 | 83.51 |
|  | NR | 24,683 | 92.75 |
| Total | Annotated | 25,769 | 96.83 |
|  | Gene | 26,613 | - |
