# Supplementary material for: Chromosome-level genome assembly of the largefin longbarbel catfish (Hemibagrus macropterus)
Source: Front Genet. 2023 Nov 1;14:1297119. doi: 10.3389/fgene.2023.1297119 (PMC10646426; doi:10.3389/fgene.2023.1297119)
Supplement: Supplementary file 7 [file Table4.docx]

**Supplementary Table S4.** Summary of BUSCO analysis results based on *Hemibagrus macropterus* gene prediction.

| Type | Number | Percent (%) |
| --- | --- | --- |
| Complete BUSCOs | 3,577 | 98.20 |
| Complete and single-copy BUSCOs | 3,499 | 96.10 |
| Complete and duplicated BUSCOs | 78 | 2.10 |
| Fragmented BUSCOs | 4 | 0.10 |
| Missing BUSCOs | 59 | 1.70 |
| Total BUSCO groups searched | 3,640 | 100.00 |
