# Supplementary material for: Chromosome-level genome assembly of the largefin longbarbel catfish (Hemibagrus macropterus)
Source: Front Genet. 2023 Nov 1;14:1297119. doi: 10.3389/fgene.2023.1297119 (PMC10646426; doi:10.3389/fgene.2023.1297119)
Supplement: Supplementary file 8 [file Table6.docx]

**Supplementary Table S6.** Contraction gene families of male *Hemibagrus macropterus* were enriched in seven KEGG pathways.

| ID | Description |
| --- | --- |
| map00603 | Glycosphingolipid biosynthesis - globo and isoglobo series |
| map05152 | Tuberculosis |
| map05150 | Staphylococcus aureus infection |
| map05168 | Herpes simplex virus 1 infection |
| map05022 | Pathways of neurodegeneration - multiple diseases |
| map04668 | TNF signaling pathway |
| map00730 | Thiamine metabolism |
